# Remaining Useful Life (RUL) Prediction Based on the Bivariant Two-Phase Nonlinear Wiener Degradation Process

**DOI:** 10.3390/e27040349

**Published:** 2025-03-27

**Authors:** Lijun Sun, Yuying Liang, Zaizai Yan

**Affiliations:** College of Science, Inner Mongolia University of Technology, Hohhot 010051, China; sunlj1015@163.com (L.S.); liangyying30@163.com (Y.L.)

**Keywords:** two-phase Wiener process, Schwarz information criterion, turbine engine, RUL prediction

## Abstract

Recent advancements in science and technology have resulted in products with enhanced reliability and extended lifespans across the aerospace and related sectors. Traditional statistical models struggle to assess their reliability accurately, prompting increased interest in predicting product lifespans during service. These products, characterized by intricate structures and diverse functionalities, exhibit complex, multistage, multiperformance, and nonlinear degradation processes. To address these challenges, this paper proposes a framework for multiperformance, multi-phase Wiener process modeling and reliability analysis. It introduces a two-phase nonlinear Wiener degradation model and identifies change points via the Schwarz information criterion (SIC). The analytical formula for remaining useful life (RUL) is obtained from the concept of the first hitting time (FHT), which considers the stochastic nature of the degradation amount at the change point. The Akaike information criterion (AIC) is then utilized, and an appropriate copula function is chosen to analyze the correlation between two performance indices, given an established complexity with parameters in the degradation model. A two-step method for estimating these uncertain parameters is presented in this paper. Validation through a turbine engine case study underscores its potential to advance reliability theory and engineering practices.

## 1. Introduction

With the continuous updating and iteration of industrial technology, high-end industrial equipment is gradually becoming larger and more complex in functionality. Compared with traditional equipment, these systems are typically integrated with multiple subsystems, such as mechanical transmission systems, motion brake systems, and electromagnetic drive systems. Industrial equipment serves as a vital tool for national development and plays a crucial role in economic construction. Malfunctions and failures in these systems can lead to significant economic impacts and social consequences, particularly in aerospace equipment. Therefore, the analysis of equipment reliability assumes paramount importance.

During equipment operation, owing to changes in the working environment and prolonged service, the components of the equipment inevitably degrade over time. This degradation eventually leads to failures that pose significant risks to personnel safety and result in substantial economic losses. For example, in July 2017, a U.S. KC-130 transport aircraft crashed, resulting in the loss of all 16 military personnel aboard. An investigation revealed that the primary cause was the degraded performance of the aircraft engine propeller [1]. Statistical studies indicate that approximately 36% of aircraft accidents in the United States between 1981 and 2001 were attributed to engine failure [2]. In China, engine failures account for an even greater proportion, approximately 60% of aircraft accidents [3]. Globally, civil aviation spends approximately 18 billion US dollars annually on repairs due to engine failure. Therefore, predicting the RUL of turbine engines is crucial for ensuring both safety and economic efficiency. Early assessment of equipment health on the basis of condition monitoring data, especially before major accidents occur, is essential for effectively predicting the RUL and preventing catastrophic failures.

Advancements in information and sensor technology have enabled the prediction of equipment RUL by monitoring degradation data and constructing stochastic models that describe the performance degradation process. This approach, rooted in probability theory, formulates the degradation law within a stochastic model framework and expresses the RUL distribution as a probability distribution. Such methods are crucial for informed decision-making in scientific maintenance, replacement strategies, and logistic support for turbine engines, making them a prominent research focus globally [4].

Choosing an appropriate stochastic model is crucial for constructing a reliable stochastic degradation model. The common models for degradation systems are primarily categorized into three types: degradation trajectory models [5], degradation amount distribution models [6], and stochastic process models [7]. Compared with the other two models, a stochastic process model can better capture uncertainty along the time axis and aligns more closely with the actual circumstances. The classical stochastic process models include the Wiener process model [8], Inverse Gaussian (IG) process model [9,10] and Gamma process model [11]. Among them, the IG process model and the Gamma process model can describe only monotonic degradation processes [12]. Owing to the influences of the surrounding environment and internal factors of the equipment, the data obtained by the sensor often exhibit nonmonotonic fluctuation characteristics. The Wiener process model, driven by Brownian motion, is a diffusion process model capable of describing many non-monotonic degradation processes [13]. It offers greater flexibility for modeling degradation measurement signals, which are widely used. For example, Zhu [14] utilized the Wiener process to model an engine and applied Bayesian theory to estimate the parameters. Son [15] employed the Wiener process to predict the lifespan of engine data. For reliability modeling and RUL analysis of an engine via stochastic processes, this paper primarily addresses this issue from the following two perspectives.

On the one hand, engines often feature complex structures and operate under harsh conditions, making them susceptible to failures and causing them to be classified as fault-prone systems. Engine failures typically stem from multiple performance aspects, which can either be independent or interdependent. If they are independent, they can be modeled separately; if they are dependent, it is necessary to establish an appropriate dependency relationship. For example, Wang’s research [16] highlighted strong correlations among various degradation performances of turbine engines, underscoring the need for comprehensive modeling approaches. Common methods used to study performance include multidimensional degradation models, degradation rate correlation models, and degradation correlation models based on the copula function [17,18]. Compared with other methods, copula function models excel in capturing nuanced correlations between degradation performances. They are widely applied in RUL prediction studies for multiperformance products because of their ability to handle both linear and nonlinear dependencies effectively, including their use in Saber Zadeh’s study for modeling multivariate degradation systems [19], and Yan’s establishment of degradation models for transmission systems and subsequent RUL predictions [20]. Zhang explored multivariate modeling via copula functions across different stochastic processes, culminating in RUL predictions [21].

On the other hand, the studies mentioned above employed only single-phase linear modeling for engine monitoring data. However, during actual engine operation, internal wear, fatigue, and corrosion gradually accumulate, potentially leading to engine failure. This does not necessarily imply immediate engine breakdown but rather results in the two-phase or even multi-phase degradation of performance, characterized by nonlinear patterns in the degradation process. Current research in this area remains limited, primarily focusing on binary nonlinear degradation issues. For example, Guo [22] utilized a binary nonlinear Wiener process to model degradation and assess the reliability of the turbofan engine lifespan. In practical scenarios involving two-phase or multi-phase nonlinear degradation models, the degradation state at change points is often stochastic and known only when a transition occurs [23]. Furthermore, it is influenced by the degradation process of the preceding phase. Addressing this issue is crucial when conducting RUL predictions.

Based on the aforementioned discussion, this paper proposes a “bivariant two-phase nonlinear Wiener process degradation model with random effects”, whose innovations are primarily reflected in the following three aspects:

(1) By combining random effects, nonlinear Wiener process models, the SIC, and copula theory, we develop a novel bivariate stage-specific degradation model. This framework simultaneously captures the synergistic degradation mechanisms of multiperformance parameters and phase-transition characteristics, addressing both dependency structures and abrupt degradation shifts. (2) Unlike existing studies that often assume continuity in degradation states at change points, we introduce the FHT concept and state transition probability at change points. This approach rigorously derives closed-form analytical expressions for the probability density function (PDF) and cumulative distribution function (CDF) of the RUL. (3) The proposed model is validated through a turbo-engine degradation case study. The results demonstrate that the Gumbel copula outperforms the traditional Gaussian copula in characterizing the dependency of turbo-engine performance parameters, providing a methodological framework for degradation modeling of complex systems. This work offers new insights into reliability prediction for multi-phase degradation processes under nonlinear and stochastic effects.

## 2. Nonlinear Multi-Phase Degradation Model and RUL Prediction

### 2.1. Nonlinear Multi-Phase Wiener Process Degradation Model

The classical Wiener process model X(t) has the following properties:

(1) $\left\{X(t),t\geq 0\right\}$ has stable independent increments, that is, $\forall t_{4}>t_{3}\geq t_{2}>t_{1}\geq 0$, and both $X(t_{2})-X(t_{1})$ and $X(t_{4})-X(t_{3})$ are mutually independent.
(2) $X(t)\sim N\left(\mu \Lambda (t),\sigma^{2}\Lambda (t)\right)$ degradation amount, where μ is the drift parameter and σ is the diffusion parameter.
(3) ∀l>0, with degradation increment $X(t+l)-X(t)\sim N\left(\mu \Delta \Lambda (t:l),\sigma^{2}\Delta \Lambda (t:l)\right)$. ΔΛ(t:l)=Λ(t+l)−Λ(t) is the increment of function Λ(t), and Λ(t) is a monotonically increasing function of time. For simplicity, let $\Lambda (t)=t^{b}$, and Λ(0)=0. In particular, ΔΛ(0:t)=Λ(0+t)−Λ(0)=Λ(t).

Therefore, the degradation process can be described as follows:

(1) $$X(t)=X(0)+\mu \Lambda (t)+\sigma B\left(\Lambda (t)\right)$$

where $B\left(\Lambda (t)\right)$ represents Brownian motion and X(0) represents the initial degradation amount, which is usually X(0)=0.

**Figure 1:**
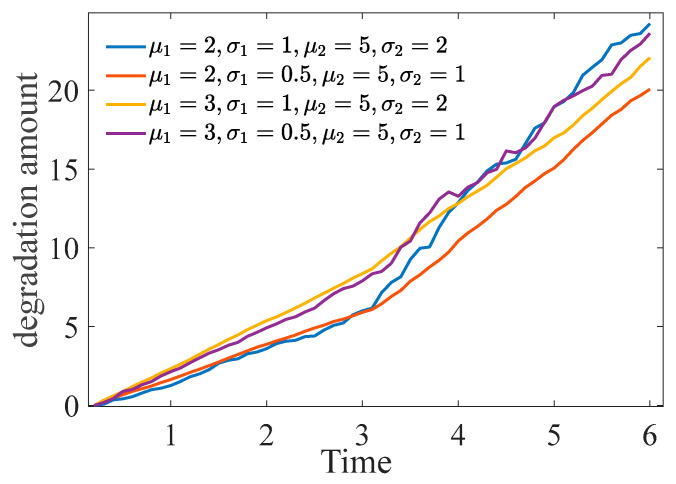
Degradation paths of linear Wiener process under different parameters.

On the basis of this model, a two-phase nonlinear Wiener degradation process with change point is expressed as follows:

(2) $$X(t)=\left\{\begin{array}{cc} X(0)+\mu_{1}\Lambda_{1}(t)+\sigma_{1}B\left(\Lambda_{1}(t)\right),\; & 0<t\leq \tau \\ X(\tau )+\mu_{2}\Lambda_{2}(t-\tau )+\sigma_{2}B\left(\Lambda_{2}(t-\tau )\right),\; & t>\tau . \end{array}\right.$$

where τ is the time at which the change point in the degradation process occurs; X(τ) is the degradation amount at the change point; and $\mu_{1},\mu_{2}$ are the drift parameters for phase I and phase II, respectively. $\sigma_{1},\sigma_{2}$ are the diffusion parameters for phase I and phase II, respectively. Figure 1 shows a two-phase nonlinear Wiener process with different parameters.

With respect to the N-phase degradation model, Equation (2) can be extended as follows:

(3) $$X(t)=\left\{\begin{array}{ll} x_{0}+\mu_{1}\Lambda_{1}(t)+\sigma_{1}B[\Lambda_{1}(t)] & ,0<t\leq \tau_{1}\\ x_{\tau_{1}}+\mu_{2}\Lambda_{2}(t-\tau_{1})+\sigma_{2}B[\Lambda_{2}(t-\tau_{1})] & ,\tau_{1}<t\leq \tau_{2}\\ \vdots & \vdots \\ x_{\tau_{n-1}}+\mu_{n}\Lambda_{n}(t-\tau_{n-1})+\sigma_{n}B[\Lambda_{n}(t-\tau_{n-1})] & ,\tau_{n-1}<t. \end{array}\right.$$

where $\mu =[\mu_{1},\mu_{2},\cdots ,\mu_{n}],\sigma =[\sigma_{1},\sigma_{2},\cdots ,\sigma_{n}]$ represent the drift coefficients and diffusion coefficients for different phases, respectively; $\Lambda (t)=[\Lambda_{1}(t),\Lambda_{2}(t-\tau_{1}),\cdots ,\Lambda_{n}(t-\tau_{n-1})]$ is the time function; and $x_{\tau }=[x_{\tau_{1}},x_{\tau_{2}},\cdots ,x_{\tau_{n-1}}]$ and $\tau =[\tau_{1},\tau_{2},\cdots ,\tau_{n-1}]$ represent the degree of degradation at the change points and at the time when the change points occur, respectively.

### 2.2. Nonlinear Multi-Phase Wiener Process Degradation Model for RUL Prediction

In practical operation, engine performance gradually degrades over time. When the degradation amount X(t) first reaches the threshold ω, engine failure is defined, and the failure time is T; thus, T represents the engine’s lifespan. Therefore, the failure time of the performance index degradation process is expressed as follows:

(4) $$T=\inf\{t:X(t)\geq \omega \left|X\right.(0)<\omega \},\;$$

where “inf” denotes the infimum.

We denote the RUL for the online operating system at moment $t_{k}$:

(5) $$L_{k}=\inf\{\left.l:X(t_{k}+l)\geq \omega \right|X(t_{k})<\omega \}.$$

On the basis of the above assumption and the FHT concept, the PDF of the RUL (l) at moment $t_{\kappa }$ and the expression for the degradation amount $x_{\kappa }$ are shown below.

Case 1: The current moment $t_{\kappa }$ is less than the change point $\tau (\tau >t_{\kappa })$:

(6) fL(l)=ω−xκ2πσ1η12(l)3exp[−ω−xκ−μ1η1(l)22σ1η12(l)]×dη1(l)dl,0<l+tκ≤τA1−B1,τ<l+tκ.

where

$$\begin{array}{ll} A_{1}= & \sqrt{\frac{1}{2\pi \Lambda_{2}^{2}(t_{\kappa }+l-\tau )\left(\sigma_{a1}^{2}+\sigma_{b1}^{2}\right)}}\exp\left[-\frac{{\left(u_{a1}-u_{b1}\right)}^{2}}{2(\sigma_{a1}^{2}+\sigma_{b1}^{2})}\right]\frac{d\Lambda_{2}(t_{\kappa }+l-\tau )}{dl}\times \\ & \left\{\frac{u_{b1}\sigma_{a1}^{2}+u_{a1}\sigma_{b1}^{2}}{\sigma_{a1}^{2}+\sigma_{b1}^{2}}\times \Phi \left(\frac{u_{b1}\sigma_{a1}^{2}+u_{a1}\sigma_{b1}^{2}}{\sqrt{\sigma_{a1}^{2}\sigma_{b1}^{2}\left(\sigma_{a1}^{2}+\sigma_{b1}^{2}\right)}}\right)+\sqrt{\frac{\sigma_{a1}^{2}\sigma_{b1}^{2}}{\sigma_{a1}^{2}+\sigma_{b1}^{2}}}\phi \left(\frac{u_{b1}\sigma_{a1}^{2}+u_{a1}\sigma_{b1}^{2}}{\sqrt{\sigma_{a1}^{2}\sigma_{b1}^{2}\left(\sigma_{a1}^{2}+\sigma_{b1}^{2}\right)}}\right)\right\}, \end{array}$$

$$\begin{array}{ll} B_{1}= & \exp\left[\frac{2\mu_{1}(\omega -x_{\kappa })}{\sigma_{1}^{2}}\right]\sqrt{\frac{1}{2\pi \Lambda_{2}^{2}(t_{\kappa }+l-\tau )\left(\sigma_{a1}^{2}+\sigma_{b1}^{2}\right)}}\exp\left[-\frac{{\left(u_{a1}-u_{b1}\right)}^{2}}{2(\sigma_{a1}^{2}+\sigma_{b1}^{2})}\right]\frac{d\Lambda_{2}(t_{\kappa }+l-\tau )}{dl}\\ & \left\{\frac{u_{c1}\sigma_{a1}^{2}+u_{a1}\sigma_{b1}^{2}}{\sigma_{a1}^{2}+\sigma_{b1}^{2}}\times \Phi \left(\frac{u_{c1}\sigma_{a1}^{2}+u_{a1}\sigma_{b1}^{2}}{\sqrt{\sigma_{a1}^{2}\sigma_{b1}^{2}\left(\sigma_{a1}^{2}+\sigma_{b1}^{2}\right)}}\right)+\sqrt{\frac{\sigma_{a1}^{2}\sigma_{b1}^{2}}{\sigma_{a1}^{2}+\sigma_{b1}^{2}}}\phi \left(\frac{u_{c1}\sigma_{a1}^{2}+u_{a1}\sigma_{b1}^{2}}{\sqrt{\sigma_{a1}^{2}\sigma_{b1}^{2}\left(\sigma_{a1}^{2}+\sigma_{b1}^{2}\right)}}\right)\right\}, \end{array}$$

$$\begin{array}{l} u_{a1}=u_{2}\Lambda_{2}(t_{\kappa }+l-\tau ),u_{b1}=\omega -x_{\kappa }-\mu_{1}[\Lambda_{1}(\tau )-\Lambda_{1}(t_{\kappa })],\\ u_{c1}=-\omega +x_{\kappa }-\mu_{1}[\Lambda_{1}(\tau )-\Lambda_{1}(t_{\kappa })],\sigma_{a1}^{2}=\sigma_{2}^{2}\Lambda_{2}(t_{\kappa }+l-\tau ),\\ \sigma_{b1}^{2}=\sigma_{1}^{2}[\Lambda_{1}(\tau )-\Lambda_{1}(t_{\kappa })],\eta_{1}(l)=\Lambda_{1}(l+t_{\kappa })-\Lambda_{1}(t_{\kappa }). \end{array}$$

**Proof.**  Suppose that the initial time is $t_{\kappa }$ and the initial degradation is $x_{\kappa }$$\left(\tau >t_{\kappa }\right)$, the expression of the two-phase nonlinear Wiener process is given as follows:

(7) $$x(t)=\left\{\begin{array}{ll} x_{k}+\mu_{1}\Lambda_{1}(t-t_{k})+\sigma_{1}B[\Lambda_{1}(t-t_{k})] & 0<t_{k}<t\leq \tau \\ x_{\tau }+\mu_{2}\Lambda_{2}(t-\tau )+\sigma_{2}B[\Lambda_{2}(t-\tau )] & t>\tau \end{array}\right..$$

If $t=l+t_{k}$, then we have

(8) $$x(l)=\left\{\begin{array}{ll} x_{k}+\mu_{1}\Lambda_{1}(l)+\sigma_{1}B[\Lambda_{1}(l)] & 0<t_{k}+l<\tau \\ x_{\tau }+\mu_{2}\Lambda_{2}(t_{k}+l-\tau )+\sigma_{2}B[\Lambda_{2}(t_{k}+l-\tau )] & t_{k}+l>\tau \end{array}\right..$$

Because the increment in the Wiener process obeys a normal distribution, the PDF of the RUL can be obtained as follows:

(9) f(l)=ω−xκ2πσ1Λ132lexp−(ω−xκ−μ1Λ1(l))22σ1Λ12(l)dΛ1(l)dl0<tk+l<τω−xτ2πσ2Λ232tk+l−τ3exp−(ω−xτ−μ2Λ2(tk+l−τ))22σ2Λ22(tk+l−τ)×dΛ2(tk+l−τ)dltk+l>τ.

□

**Lemma 1**  **([24]).** *If* $x(t)=\mu t+\sigma_{B}B(t)$* represents a linear Wiener process starting from 0, according to the Fokker–Planck equation (Kolmogorov equation), the transition density function with*ω*as the threshold can be derived as follows:*

g(x,t)=12πtσB2exp−x−μt22σBt2−exp(2μωσB2)exp−x−2ω−μt22σBt2.

For this study, x needs to be converted to a form with an initial value of 0:

$$X(l)=x(l)-x_{k}=\mu_{1}\Lambda_{1}(l)+\sigma_{1}B[\Lambda_{1}(l)]\;0<l\leq \tau -t_{k}$$

At this time, the threshold corresponds to $\omega_{k}=\omega -x_{k}$. In this case, the transition PDF with threshold $\omega_{k}$ is in the form of

gτ(xk,τ)=12πσ12Λ1(τ−tκ)exp−x(τ−tk)−μ1Λ1(τ−tκ)22σ1Λ12(τ−tκ)−exp(2μ(ω−xk)σB2)exp−xτ−2(ω−xk)−μ1Λ1(τ−tκ)22σ1Λ12(τ−tκ).

According to the Bayesian theorem, the PDF of the two-phase nonlinear Wiener process, fully accounting for the stochastic nature of change point degradation, is as follows:

(10) $$f_{L}(l)=\left\{\begin{array}{ll} \frac{\omega -x_{k}}{\sqrt{2\pi \sigma_{1}^{2}\Lambda_{1}^{3}(l)}}\exp\left[-\frac{{\left(\omega -x_{k}-\mu_{1}\Lambda_{1}(l)\right)}^{2}}{2\sigma_{1}^{2}\Lambda_{1}(l)}\right]\frac{d\Lambda_{1}(l)}{dl} & 0<t_{k}+l<\tau \\ \int_{-\infty }^{\omega }\frac{\omega -x_{\tau }}{\sqrt{2\pi \sigma_{2}^{2}\Lambda_{2}^{3}(t_{k}+l-\tau )}}\exp\left[-\frac{{\left(\omega -x_{\tau }-\mu_{2}\Lambda_{2}(t_{k}+l-\tau )\right)}^{2}}{2\sigma_{2}^{2}\Lambda_{2}(t_{k}+l-\tau )}\right]\\ \frac{d\Lambda_{2}(t_{k}+l-\tau )}{dl}\cdot g_{\tau }(x_{\tau })dx_{\tau } & t_{k}+l>\tau \end{array}\right..$$

Case 2: The current time $t_{\kappa }$ is greater than the change point $\tau (\tau \leq t_{\kappa })$:

(11) fL(l)=ω−xκ2πσ2η22l3exp−(ω−xκ−μ2η2(l))22σ2η22(l)dη2(l)dl, 

where $\eta_{2}(l)=\Lambda_{2}(l+t_{\kappa }-\tau )-\Lambda_{2}(t_{\kappa }-\tau )$. ϕ(⋅) represents the PDF and Φ(⋅) represents the CDF of the standard normal distribution. Since case 2 with τ>0 and $t_{\kappa }=0,$ will not appear in the PDF of the RUL, specific expressions for the RUL under a single performance can be obtained.

## 3. Reliability Analysis of the Bivariate Two-Phase Nonlinear Wiener Process

### Copula Function Theory

The copula function is a method in multivariate statistics and correlation analysis. It serves as a crucial link between the joint distribution function (JDF) and the marginal distribution function (MDF). The copula function was first introduced by Sklar in 1959, and its theorem is as follows:

Let H(⋅,⋅) be a JDF, and F(⋅) and G(⋅) be the MDFs of the variables. Then, a copula function C(⋅,⋅) must exist that is satisfied for all $x^{\left(1\right)}$ and $x^{\left(2\right)}$ in $\left[-\infty ,+\infty \right]$:

(12) $$H(x^{\left(1\right)},x^{\left(2\right)})=C(F(x^{\left(1\right)}),G(x^{\left(2\right)});\theta ),\;$$

where θ is the relevant parameter of the copula function. If $F\left(x^{\left(1\right)}\right)$ and $G\left(x^{\left(2\right)}\right)$ are continuous, C(⋅,⋅) will be the only one; if not, C(⋅,⋅) is not the only one for certain. The above formula can be used to solve the JDF from the MDF. On this basis, the MDF and copula correlation function can be studied separately. Additionally, by finding the derivatives of the two sides of Equation (12) in terms of x and y, we can obtain the PDF corresponding to the JDF $H(x^{\left(1\right)},x^{\left(2\right)})$.

(13) $$h(x^{\left(1\right)},x^{\left(2\right)})=c(F(x^{\left(1\right)}),G(x^{\left(2\right)});\theta )f\left(x^{\left(1\right)}\right)g\left(x^{\left(2\right)}\right).$$

The PDF of the copula function may be further deduced as follows:

(14) $$c(F(x^{\left(1\right)}),G(x^{\left(2\right)});\theta )=\frac{\partial C(F(x^{\left(1\right)}),G(x^{\left(2\right)});\theta )}{\partial F(x^{\left(1\right)})\partial G(x^{\left(2\right)})},\;$$

where $c(F(x^{\left(1\right)}),G(x^{\left(2\right)});\theta )$ is the PDF of the copula function $C(F(x^{\left(1\right)}),G(x^{\left(2\right)});\theta )$, and $f\left(x^{\left(1\right)}\right)$ and $g\left(x^{\left(2\right)}\right)$ are the PDFs of the MDFs $F\left(x^{\left(1\right)}\right)$ and $G\left(x^{\left(2\right)}\right)$, respectively.

On the basis of commonly used assumptions [25], this paper constructs a correlation between engine performances via the copula function. It ignores the correlation between the two performances in different time intervals and considers only the correlation between the engine performances within the same time interval. It assumes that the degradation of the two performances is represented by $X^{\left(1\right)}$ and $X^{\left(2\right)}$, with the respective MDFs $F(X^{\left(1\right)})$ and $G(X^{\left(2\right)})$. Therefore, according to the correlation theory of the copula function, the JDF $H(X^{\left(1\right)},X^{\left(2\right)})$ of the two engine performances is as follows:

(15) $$H(X^{\left(1\right)},X^{\left(2\right)})=C\left(F(X^{\left(1\right)}),G(X^{\left(2\right)});\theta \right).$$

Four common copula functions are given in this paper (see Table 1).

The reliability is calculated under the assumption that the failure times of the two performance characteristics of the engine are $T^{\left(1\right)}$ and $T^{\left(2\right)}$.

(16) $$\begin{array}{ll} R(t) & =P(T^{\left(1\right)}>t,T^{\left(2\right)}>t)=1+P(T^{\left(1\right)}\leq t,T^{\left(2\right)}\leq t)-P(T^{\left(1\right)}\leq t)-P(T^{\left(2\right)}\leq t)\\ & =R^{\left(1\right)}(t)+R^{\left(2\right)}(t)-1+C(F(t),G(t);\theta ). \end{array}$$

There are many choices available for the copula function, which results in different outcomes. Choosing the appropriate copula function is crucial according to the actual situation. The AIC is a criterion for evaluating the complexity of statistical models and assessing their goodness of fit. The primary principle of the AIC is to balance the model’s fit to the data while minimizing its complexity. Specifically, the AIC favors models with lower values, as this indicates a more effective equilibrium between data fitting and prevention of overfitting; so, this paper suggests the use of the AIC to select the appropriate copula function for reliability modeling.

(17) $$AIC=-2\ln L(\hat{\theta })+2m,\;$$

where $L(\hat{\theta })$ represents the likelihood function for the proposed model, and m represents the number of unknown parameters in the likelihood function. The smaller the AIC, the better the model; typically, the model with the smallest AIC is selected.

## 4. Parameter Estimation

Suppose *N* products participate in the test, with *M* measurements made for each product. Each measurement includes two performance indicators, corresponding to *N* sets of degradation data. Let $x_{i,j}^{\left(k\right)}$ denote the k-th (k=1,2) amount of degradation for the i-th (i=1,2,⋯N) sample at the j-th (j=1,2,⋯M) moment $t_{i,j}$. The observed degradation increment is $\Delta x_{i,j}^{\left(k\right)}=x_{i,j}^{\left(k\right)}-x_{i,j-1}^{\left(k\right)}$, and the initial moment $t_{i,0}^{\left(k\right)}=0,x_{i,0}^{\left(k\right)}=0$ is set.

Assuming that change point time is known, values are only collected at the sampling moments, i.e., $\tau_{i}^{\left(k\right)}\in \left\{t_{i,0},t_{i,1},\cdots ,t_{i,m}\right\}$. Then, $\Delta x_{1i,j}^{\left(k\right)}=\left\{x_{i,0}^{\left(k\right)},x_{i,1}^{\left(k\right)},\cdots ,x_{i,\tau_{i}^{\left(k\right)}}^{\left(k\right)}\right\}$ denotes the first-phase degradation data, corresponding to the value of the sampling moment $\Delta t_{1i,j}^{\left(k\right)}\left\{t_{i,0},t_{i,1},\cdots ,t_{i,\tau_{i}^{\left(k\right)}}\right\}$. Then, $\Delta x_{2i,j}^{\left(k\right)}=\left\{x_{i,\tau_{i+1}^{\left(k\right)}}^{\left(k\right)},x_{i,\tau_{i+2}^{\left(k\right)}}^{\left(k\right)},,\cdots ,x_{i,m}^{\left(k\right)}\right\}$ denotes the two-phase degradation data, and the corresponding sampling moment value is $\Delta t_{2i,j}^{\left(k\right)}\left\{t_{i,\tau_{i+1}^{\left(k\right)}},t_{i,\tau_{i+2}^{\left(k\right)}},\cdots ,t_{i,m}\right\}$.

**Figure 2:**
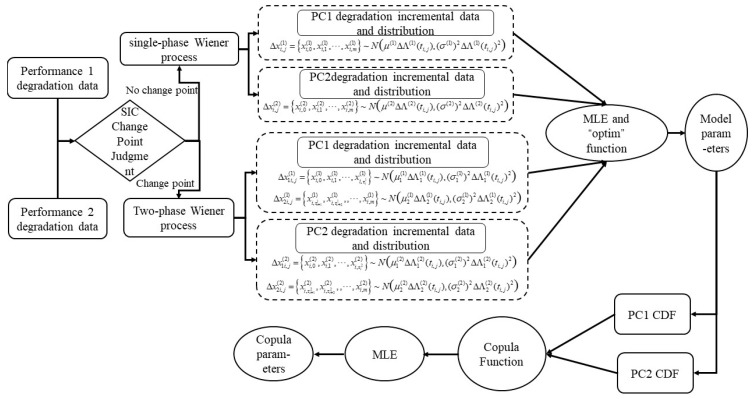
Path diagram of the parameter estimation algorithm.

In this study, the complexity of the expression increases because of the unknown parameters of the binary-stage Wiener process. Consequently, the use of traditional parameter estimation methods not only results in high computational intensity but also challenges the accurate determination of model parameters. To address this issue, a function inference method based on marginal distribution is presented. The central idea of the method is twofold: First, separate parameter estimation is conducted for the marginal distribution; then, further estimation of the copula parameters is performed. Second, degradation data from a single-performance two-phase nonlinear Wiener process are utilized to monitor points of unknown variables before advancing the parameter estimation. Figure 2 illustrates the parameter estimation pathway for the binary-stage Wiener process.

### 4.1. Schwartz Information Criterion (SIC)

Schwartz introduced the SIC in 1978, which is derived from the AIC and takes a Bayesian perspective. The SIC serves as a discriminative criterion for assessing the presence of a change point in a model via its log-likelihood function. The principle behind the SIC is that if a change point exists in the sequence under consideration, the entropy of its samples will differ significantly from that of samples without a change point. This criterion proves effective in change point detection [26]. Its expression is as follows:

$$\mathrm{SIC}=-2\log L(\hat{\theta })+q\log p$$

where $L(\hat{\theta })$ represents the likelihood function for the proposed model; $\hat{\theta }$ represents the estimate value of the parameter θ; *q* represents the count of parameters in this model; and *p* represents the count of samples. According to the SIC, the following assumptions are made:

Assumption $H_{0}$: Equal values for each parameter indicate the absence of change points in the model. Its corresponding $SIC_{i}(m)$ value is as follows:

(18) $$SIC_{i}(m)=m\ln2\pi +m\ln\sum_{j=1}^{m}{\left(\Delta x_{i,j}-\bar{\Delta x_{i}}\right)}^{2}+m+(2-m)\ln m,\;$$

where $\bar{\Delta x_{i}}=\frac{1}{m}\sum_{j=1}^{m}\Delta x_{i,j}$.

Assumption $H_{1}$: There exists a change point τ. Stages before τ degrade according to $X_{1}(t;\mu_{1},\sigma_{1}^{2})$, and stages after τ degrade according to $X_{2}(t;\mu_{2},\sigma_{2}^{2})$. Its $SIC_{i}(s)$ value is as follows:

(19) $$SIC_{i}(s)=m\ln2\pi +n\ln\frac{1}{n}\sum_{j=1}^{s}{\left(\Delta x_{i,j}-\bar{\Delta x_{1i}}\right)}^{2}+4\ln m+(m-n)\ln\frac{1}{m}\sum_{j=s+1}^{m}{\left(\Delta x_{i,j}-\bar{\Delta x_{2i}}\right)}^{2}-m.$$

where $\bar{\Delta x_{1i}}=\frac{1}{s}\sum_{j=1}^{s}\Delta x_{i,j}$, $\bar{\Delta x_{2i}}=\frac{1}{m-s}\sum_{j=s+1}^{m}\Delta x_{i,j}$.

According to the SIC, if $SIC_{i}(m)>\underset{2<s\leq m-2}{\min}SIC_{i}(s)$, then $H_{0}$ is rejected because of a variation point, where the estimate of the variation point $\hat{\tau }=\hat{s}$ is as follows:

$$SIC_{i}(\hat{s})=\underset{2<s\leq m-2}{\min}SIC_{i}(s).$$

Using the SIC method, the change point of performance can be estimated via degradation monitoring data. The specific calculation process is shown in Algorithm 1.
**Algorithm 1.** Calculation of Change Point Occurrence in Degradation Process
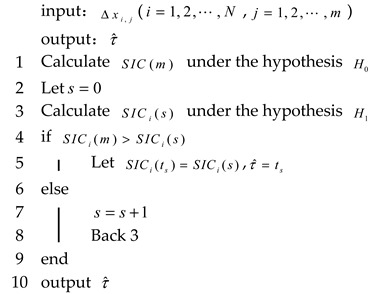


### 4.2. Estimation of Unknown Parameters

According to the SIC, the model’s variable point values can be obtained. On this basis, taking into account the incremental independence of the Wiener process increments, $\Delta x_{i,j}^{\left(k\right)}$ follows a multivariate normal distribution, and the two-phase degradation process can be regarded as two independent single-phase degradation processes.

The average of phases I and II can be represented as

$${\tilde{\mu }}_{1,i}=\mu_{1,i}T_{1,i}\;;{\tilde{\mu }}_{2,i}=\mu_{2,i}T_{2,i}$$

where $T_{1,i}=\left\{t_{i,0},t_{i,1},\cdots ,t_{i,\tau_{i}}\right\}$, and $T_{2,i}=\left\{t_{i,\tau_{i+1}},t_{i,\tau_{i+2}},\cdots ,t_{i,m}\right\}$. The variance of phases I and II can be represented as

$$\Sigma_{1,i}=\sigma_{1,i}^{2}Q_{1,i};\Sigma_{2,i}=\sigma_{2,i}^{2}Q_{2,i},$$

where $Q_{1,i}=\left[\begin{array}{cccc} \Lambda_{1}(t_{i,1}) & \Lambda_{1}(t_{i,1}) & \cdots & \Lambda_{1}(t_{i,1})\\ \Lambda_{1}(t_{i,1}) & \Lambda_{1}(t_{i,2}) & \cdots & \Lambda_{1}(t_{i,2})\\ \vdots & \vdots & \ddots & \vdots \\ \Lambda_{1}(t_{i,1}) & \Lambda_{1}(t_{i,2}) & \cdots & \Lambda_{1}(t_{i,{\tilde{t}}_{i}}) \end{array}\right];$

$$Q_{2,i}=\left[\begin{array}{cccc} \Lambda_{2}(t_{i,{\tilde{\tau }}_{i}+1}-t_{i,{\tilde{\tau }}_{i}}) & \Lambda_{2}(t_{i,{\tilde{\tau }}_{i}+1}-t_{i,{\tilde{\tau }}_{i}}) & \cdots & \Lambda_{2}(t_{i,{\tilde{\tau }}_{i}+1}-t_{i,{\tilde{\tau }}_{i}})\\ \Lambda_{2}(t_{i,{\tilde{\tau }}_{i}+1}-t_{i,{\tilde{\tau }}_{i}}) & \Lambda_{2}(t_{i,{\tilde{\tau }}_{i}+2}-t_{i,{\tilde{\tau }}_{i}}) & \cdots & \Lambda_{2}(t_{i,{\tilde{\tau }}_{i}+2}-t_{i,{\tilde{\tau }}_{i}})\\ \vdots & \vdots & \ddots & \vdots \\ \Lambda_{2}(t_{i,{\tilde{\tau }}_{i}+1}-t_{i,{\tilde{\tau }}_{i}}) & \Lambda_{2}(t_{i,{\tilde{\tau }}_{i}+2}-t_{i,{\tilde{\tau }}_{i}}) & \cdots & \Lambda_{2}(t_{i,m_{i}}-t_{i,{\tilde{\tau }}_{i}}) \end{array}\right].$$

In turn, the parameter $\theta_{i}=(\mu_{1,i},\mu_{2,i},\sigma_{1,i},\sigma_{2,i},b_{1,i},b_{2,i}{)}^{\prime }$ can be estimated, where $b_{1,i}$ and $b_{2,i}$ represent the parameters of the nonlinear form. The log-likelihood function of the model is formulated as follows:

(20) $$\begin{array}{ll} \ln L(\mu_{1,i},\mu_{2,i},\sigma_{1,i},\sigma_{2,i},b_{1,i},b_{2,i}|X_{i})= & -\frac{1}{2}\ln(2\pi )\tau_{i}-\frac{1}{2}\ln\left|\Sigma_{1,i}\right|-\frac{1}{2}{\left(X_{1,i}-{\tilde{\mu }}_{1,i}\right)}^{\prime }\Sigma_{1,i}^{-1}\left(X_{1,i}-{\tilde{\mu }}_{1,i}\right)\\ & -\frac{1}{2}\ln(2\pi )(m_{i}-\tau_{i})-\frac{1}{2}\ln\left|\Sigma_{2,i}\right|-\frac{1}{2}{\left(X_{2,i}-{\tilde{\mu }}_{2,i}\right)}^{\prime }\Sigma_{2,i}^{-1}\left(X_{2,i}-{\tilde{\mu }}_{2,i}\right). \end{array}$$

Next, let us obtain the first-order partial derivatives of *m* and *n* in the log-likelihood function:

(21) $$\frac{\partial \ln L(\theta_{i}|X_{i})}{\partial \mu_{1,i}}=T_{1,i}^{\prime }\Sigma_{1,i}^{-1}X_{1,i}-\mu_{1,i}T_{1,i}^{\prime }\Sigma_{1,i}^{-1}T_{1,i},\;$$

(22) $$\frac{\partial \ln L(\theta_{i}|X_{i})}{\partial \mu_{2,i}}=T_{2,i}^{\prime }\Sigma_{2,i}^{-1}X_{2,i}-\mu_{2,i}T_{2,i}^{\prime }\Sigma_{2,i}^{-1}T_{2,i}.$$

Let both derivatives be equal to zero; then, the MLE values of $\mu_{1,i}$ and $\mu_{2,i}$ are as follows:

(23) $$\mu_{1,i}=\frac{T_{1,i}^{\prime }\Sigma_{1,i}^{-1}X_{1,i}}{T_{1,i}^{\prime }\Sigma_{1,i}^{-1}T_{1,i}},\;\mu_{2,i}=\frac{T_{2,i}^{\prime }\Sigma_{2,i}^{-1}X_{2,i}}{T_{2,i}^{\prime }\Sigma_{2,i}^{-1}T_{2,i}}$$

By substituting the values of $\mu_{1,i}$ and $\mu_{2,i}$ into the MLE function, the contour likelihood function of $\sigma_{1,i},\sigma_{2,i},b_{1,i}$ and $b_{2,i}$ can be obtained:

(24) $$\begin{array}{ll} \ln L(\sigma_{1,i},\sigma_{2,i},b_{1,i},b_{2,i}|\mu_{1,i},\mu_{2,i},X_{i})= & -\frac{1}{2}\ln(2\pi )\tau_{i}-\frac{1}{2}\ln\left|\Sigma_{1,i}\right|\\ & -\frac{1}{2}(X_{1,i}-\frac{T_{1,i}^{\prime }\Sigma_{1,i}^{-1}X_{1,i}}{T_{1,i}^{\prime }\Sigma_{1,i}^{-1}T_{1,i}}T_{1,i}{)}^{\prime }\Sigma_{1,i}^{-1}(X_{1,i}-\frac{T_{1,i}^{\prime }\Sigma_{1,i}^{-1}X_{1,i}}{T_{1,i}^{\prime }\Sigma_{1,i}^{-1}T_{1,i}}T_{1,i})\\ & -\frac{1}{2}\ln(2\pi )(m_{i}-\tau_{i})-\frac{1}{2}\ln\left|\Sigma_{2,i}\right|\\ & -\frac{1}{2}(X_{2,i}-\frac{T_{2,i}^{\prime }\Sigma_{2,i}^{-1}X_{2,i}}{T_{2,i}^{\prime }\Sigma_{2,i}^{-1}T_{2,i}}T_{2,i}{)}^{\prime }\Sigma_{2,i}^{-1}(X_{2,i}-\frac{T_{2,i}^{\prime }\Sigma_{2,i}^{-1}X_{2,i}}{T_{2,i}^{\prime }\Sigma_{2,i}^{-1}T_{2,i}}T_{2,i}). \end{array}$$

For the two-phase degradation model, the unknown parameters $\sigma_{1,i},\sigma_{2,i},b_{1,i},b_{2,i}$ can be solved via an optimization algorithm. In this paper, we utilize the “optim” optimization function in R to solve for the unknown parameters $\sigma_{1,i},\sigma_{2,i},b_{1,i},b_{2,i}$ of the model. The estimated parameters are substituted into Equation (23) to obtain the estimates of $\mu_{1,i},\;\mu_{2,i}$. By substituting all parameters into Equation (10), the PDF of a single performance characteristic, denoted as $f_{1}\left(x_{i,j}^{\left(1\right)}\right)$, is obtained. Integrating $f_{1}\left(x_{i,j}^{\left(1\right)}\right)$ provides the corresponding CDF for this performance characteristic, labeled as $F_{1}\left(x_{i,j}^{\left(1\right)}\right)$. Repeating the above steps for the second performance characteristic yields its CDF ($F_{2}\left(x_{i,j}^{\left(2\right)}\right)$) and PDF ($f_{2}\left(x_{i,j}^{\left(2\right)}\right)$).

Based on the aforementioned parameter estimation framework, the correlation parameters θ in the copula function can be estimated, and the overall log-likelihood function is given as follows:

(25) $$\ln L(\theta )=\sum_{i=1}^{N}\sum_{j=1}^{m}\ln c\left(F_{1}\left(x_{i,j}^{\left(1\right)}\right),F_{2}(x_{i,j}^{\left(2\right)});\theta \right)+\sum_{i=1}^{N}\sum_{j=1}^{m}\sum_{k=1}^{2}f_{\left(k\right)}(x_{i,j}^{\left(k\right)}).$$

Taking the first derivative of the equation above yields the maximum likelihood estimate (MLE) of parameter θ. The specific algorithmic flowchart for the aforementioned parameter estimation procedure is illustrated in Algorithm 2.
**Algorithm 2.** Calculation of Unknown Parameters in Degradation Process
Input: $\tau_{i},\Delta x_{i,j}^{\left(1\right)},\Delta x_{i,j}^{\left(2\right)}$(i=1,2,⋯,N, j=1,2,⋯,m)
Output: $\mu_{1,i}^{\left(k\right)},\;\mu_{2,i}^{\left(k\right)},\sigma_{1,i}^{\left(k\right)},\sigma_{2,i}^{\left(k\right)},b_{1,i}^{\left(k\right)},b_{2,i}^{\left(k\right)},\theta ,(k=1,2)$
1Let $\Delta x_{i,j}^{\left(1\right)}=\Delta x_{i,j}$
2$\Delta x_{1i,j}=\Delta x_{i,j}(i=1,\cdots ,N;j=1,\cdots ,\tau_{i});\Delta x_{2i,j}=\Delta x_{i,j}(i=1,\cdots ,N;j=\tau_{i}+1,\cdots ,m)$3The parameters in Equation (24) are computed using the “optim” function in R 4.4.2.4Calculated to obtain $\sigma_{1,i},\sigma_{2,i},b_{1,i},b_{2,i}$
5Parameters $\mu_{1,i},\;\mu_{2,i}$ are calculated using Equation (23)6Substituting back Equation (10) to obtain the PDF $f_{1}\left(x_{i,j}^{\left(1\right)}\right)$ of the model7Integrating to obtain the CDF $F_{1}\left(x_{i,j}^{\left(1\right)}\right)$
7Repeat steps 1–7 to get another performance PDF $f_{2}(x_{i,j}^{\left(2\right)})$ and CDF $F_{2}\left(x_{i,j}^{\left(2\right)}\right)$
8Parameter θ is obtained via MLE using Equation (25)9output $\mu_{1,i}^{\left(k\right)},\;\mu_{2,i}^{\left(k\right)},\sigma_{1,i}^{\left(k\right)},\sigma_{2,i}^{\left(k\right)},b_{1,i}^{\left(k\right)},b_{2,i}^{\left(k\right)},\theta ,(k=1,2)$


## 5. Example Analysis

### 5.1. Experimental Data Sources

**Figure 3:**
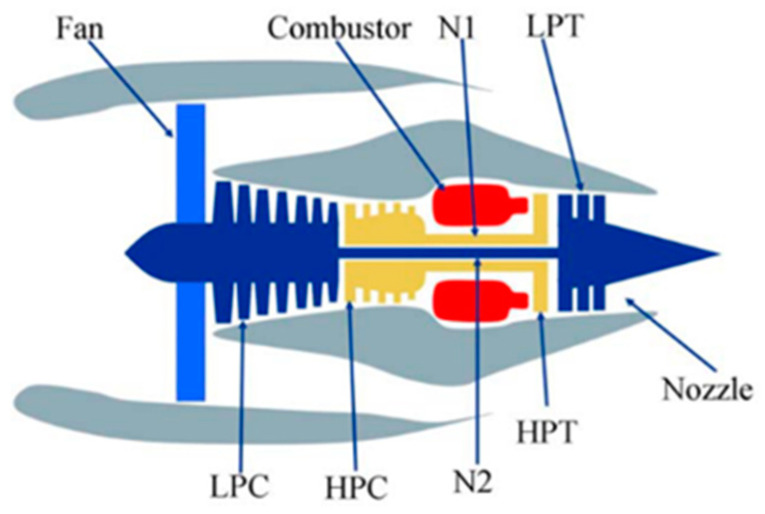
Sketch of C-MAPSS simulated engine.

The RUL prediction algorithm described here was applied to simulate experiments via a degraded dataset of turbofan aero-engines provided by NASA. This dataset was derived from C-MAPSS and models a two-shaft turbofan engine [26]. The dataset also incorporates a power management system that varies engine thrust levels across different conditions of flight. The structure of the engine is illustrated in Figure 3.

In this study, we selected the FD001 dataset for analysis and study. This subset comprises data from 100 engines, totaling more than 33,000 flight cycles of engine status data. Each engine dataset contains 26 columns, including the engine number, number of operating cycles, three sensor operating settings, and 21 sensor measurements (See Table 2) [27].

The performance degradation data detected by the sensors originate from various components of the turbine engine, which exhibit significant differences in units of measurement and scales. These variations in degradation data can impact the outcomes of modeling and analysis, necessitating data normalization. Data normalization methods involve mathematical transformations that map data to a specific range, mitigating differences in scale across datasets. In this study, we employed the mean normalization method to preprocess the data. Furthermore, Spearman’s correlation coefficient assesses the correlation between performance degradation and operating time [28]. This helps identify and filter out performance trends characterized by significant degradation.

The larger the Spearman’s rank correlation coefficient, the more pronounced the monotonic degradation trend of performance over time. Conversely, the closer the Spearman’s rank correlation coefficient is to 0, the less evident the performance degradation trend becomes as the engine running time increases, indicating a lack of significant degradation [29]. In this work, we analyzed Spearman’s rank correlation coefficients to assess the degradation trends of the 21 sensors. The results are presented in Table 3.

### 5.2. Reliability Analysis

On the basis of the analysis of engine performance degradation monotonicity above, degradation trend values for different sensors were determined. In this study, a threshold greater than 0.8 was generally chosen as an experimental parameter. To further illustrate the efficacy of the presented method, two sets of sensor data with trend values above 0.9 were selected for analysis: Sensor No. 9 and Sensor No. 14 (refer to Figure 3).

#### 5.2.1. Data Denoising

Owing to the complex internal environment of turbine engines during operation, monitored data often contain significant noise. This noise can obscure accurate reflection of the engine’s state. Therefore, in an RUL study using sensor data, initial data filtering is essential.

**Figure 4:**
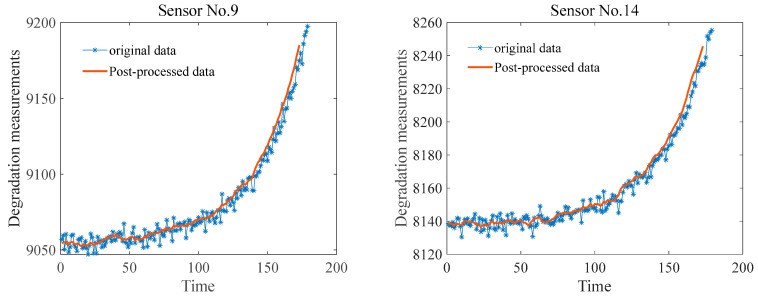
Comparison of sliding average filtering processing results for data from Sensors 9 and 14.

Sliding average filtering is a straightforward and effective technique for filtering noisy data. This method involves sliding a window across the data along the time axis. Within each window, the data values are averaged, effectively smoothing the data and reducing the impact of outliers. Owing to its efficacy, sliding average filtering is widely employed across various fields. The sliding average filtering method was used to process data from Sensor No. 9 and Sensor No. 14. The process results are illustrated in Figure 4.

#### 5.2.2. Engine Reliability Analysis

**Figure 5:**
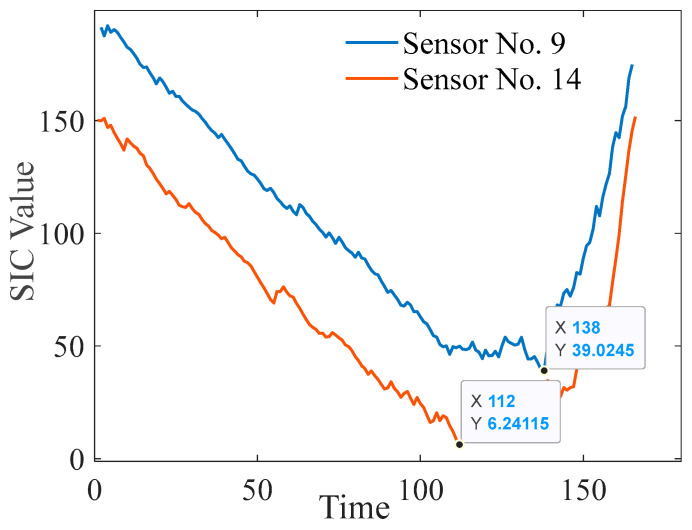
Plot of two performance SIC values over time.

As illustrated in Figure 4, the phases and nonlinearity of the degradation data are clearly evident. In this study, for reliability modeling analysis via the binary two-phase nonlinear Wiener process, the initial step involved determining the change point in engine performance degradation. Hence, the SIC was used to identify the location of the variation point, and the results are presented in Figure 5.

Figure 5 clearly shows that the locations of the variation points calculated via the SIC corresponds to the locations of the two phases of change, confirming the accuracy of change point detection. Furthermore, using the parameter estimation method presented in this paper, we identified the unknown parameters in the degradation model for Sensor No. 9 and Sensor No. 14. The specific values are presented in Table 4.

**Figure 6:**
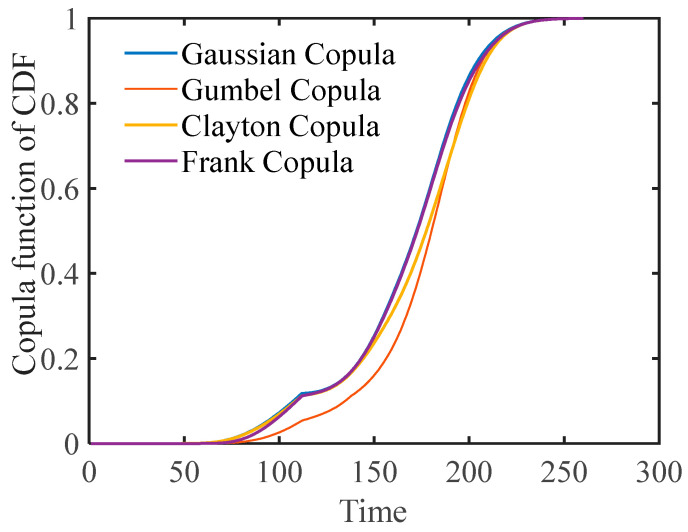
Plot of CDF with different Copula functions.

As observed from the table above, there is a notable difference between the first- and second-phase model parameters for both Sensor No. 9 and Sensor No. 14. This disparity indicates varying degradation rates across different phases of the same performance degradation, thereby validating the use of two-phase performance modeling in this study. Furthermore, the corresponding parameters were computed via different copula functions (Table 5). The copula parameters demonstrate a clear positive correlation between the two performances, underscoring the validity of the proposed method. Cumulative distribution function plots under the four copula functions were also generated (see Figure 6).

To compare the effectiveness of each model fit, the corresponding AIC values are provided in Table 5.

The above table shows that the AIC value of the Gumbel copula is clearly lower compared to that of the other three copula functions. On the basis of these goodness-of-fit metrics, we can infer that the Gumbel copula is more appropriate for describing the degradation modeling of the turbine engine.

**Figure 7:**
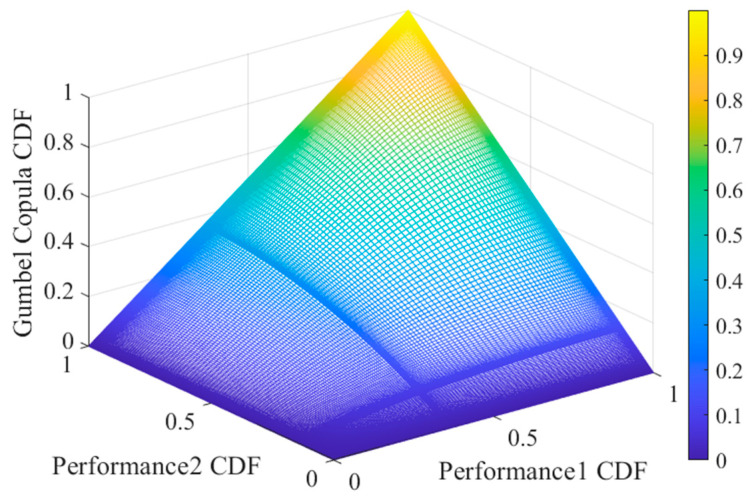
Plot of CDF for turbine engine.

Figure 6 clearly shows that if the other three copula functions are used for modeling and analysis, the predicted model’s failure time will occur earlier. Therefore, the Gumbel copula was selected to construct the cumulative distribution function of turbine engine performance, as illustrated in Figure 7.

For ease of subsequent analyses, we denote the models as follows: M1 for the binary Wiener process model using the Gumbel copula function, M2 for sensor degradation model #9, M3 for sensor degradation model #14, and M4 for the performance-independent degradation model. The corresponding AIC values for these four models are provided in Table 6.

**Figure 8:**
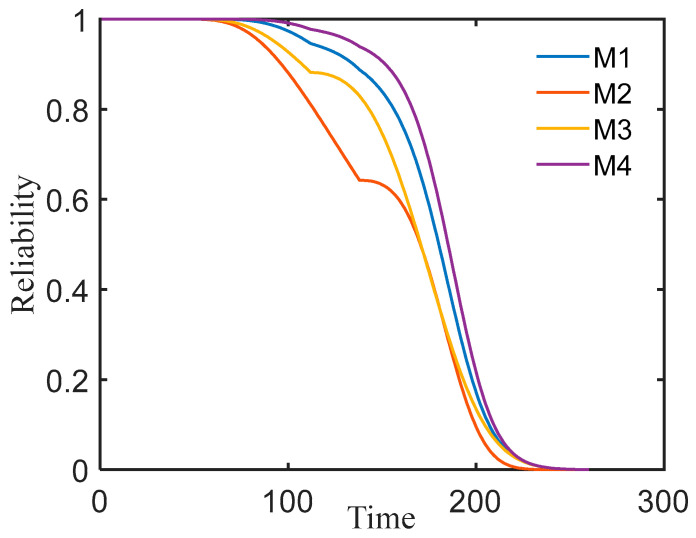
Plot of the four models’ reliability functions.

The reliability functions under the four models were obtained. The results are shown in Figure 8 below.

Figure 8 clearly shows that all four models exhibit distinct two-phase characteristics. According to Table 5, the methodology presented in this paper is more appropriate for turbine engine performance failure problems. Under different reliability thresholds, M1 demonstrates superior balance and accuracy in RUL prediction. For example, in high-reliability scenarios (R = 0.8), M1 predicts the RUL as 84 cycles, showing reductions of 33.9% and 13.4% compared to M2 (127 cycles) and M3 (97 cycles), respectively. This highlights its sensitivity to early degradation signals, effectively avoiding maintenance delays from over-optimistic predictions. Under low-reliability critical conditions (R = 0.1), M1 predicts the RUL as 33 cycles, with reductions of 19.5% and 8.3% compared to M2 (41 cycles) and M3 (36 cycles), demonstrating a reduced risk of late-stage misjudgment near failure. Compared with M4, M1 extends the RUL prediction by 13.5% (84 cycles vs. M4’s 74 cycles) under high reliability, minimizing premature maintenance. Meanwhile, it reduces prediction deviation under low reliability (33 cycles vs. M4’s 31 cycles, 6.5% error reduction), resolving M4’s systematic contradictions (early over-conservatism and late over-aggressiveness) caused by neglecting parameter correlations.

In conclusion, incorrect modeling choices can significantly impact the reliability assessment of the engine, resulting in divergent outcomes. The proposed method underscores the utility of copula functions in integrating diverse performance data to increase the accuracy and reliability of RUL predictions in complex systems such as turbine engines. By leveraging such advanced modeling techniques, engineers and researchers can better anticipate and mitigate potential failures, thereby improving overall operational efficiency and safety.

## 6. Conclusions and Recommendations

Field reliability represents a product’s reliability as demonstrated in real-world environments. With advancements in sensor technology, substantial amounts of degradation data can now be collected, offering crucial support for modeling and analyzing product field reliability. This is particularly significant for high-precision products such as turbine engines. This paper focuses on the operational aspects of turbine engines, using the turbine engine dataset from NASA as a case study. Considering its variable operating conditions, complex structure, and diverse performance characteristics, we utilized Spearman’s rank correlation coefficient to identify significant performance degradation trends. Specifically, the degradation data from Sensor No. 9 and Sensor No. 14 were selected for comprehensive reliability analysis. The main conclusions reached in this study are listed below:

(1) Considering that the degradation amount at the change point is a random variable, a new reliability analysis method for a two-phase nonlinear Wiener process is proposed. This approach is more general and aligns better with engineering practice.
(2) The CDF and PDF of the single-performance two-phase nonlinear Wiener process are derived under the definition of FHT. Copula functions are employed to connect the two performances, yielding joint probability density functions and cumulative distribution functions that enable real-time estimation of the RUL.
(3) For the proposed model, which involves multiple unknown parameters, this paper introduces a two-step parameter estimation method. Initially, parameter estimation was performed, and then the SIC was used to accurately determine the change point during the degradation of turbine engine performance. This approach circumvents the challenge of directly estimating numerous model parameters via maximum likelihood estimation.
(4) An analysis of the turbine engine example revealed that the Gumbel copula is better suited for describing turbine engine degradation modeling than the other three copula functions. This paper includes cumulative distribution function plots of the turbine engine based on the Gumbel copula.
(5) The research presented in this paper offers valuable guidance for future investigations into the degradation of complex systems. Initially, it employs Spearman’s rank correlation coefficient to identify the primary performance metrics impacting reliability. This method models these metrics individually and uses the SIC method to detect potential change points within the performance data, thereby facilitating the selection of suitable degradation models. Finally, it establishes the interdependencies between different performance metrics through the copula function, enabling a comprehensive analysis of the RUL of complex products.

While this study can address some practical engineering issues, it still has certain limitations. Future research will focus on the following directions: (1) Intelligent reliability modeling for small-sample scenarios. To tackle the widespread challenge of small-sample data in engineering, transferable deep reinforcement learning (DRL) (e.g., the composite material lifespan prediction framework proposed by Liu et al. [30]) will be integrated with degradation stochastic process theory, constructing a DRL-based degradation modeling system with strong generalization capabilities. (2) Dynamic analysis framework driven by random variation points. Future research should overcome the limitations of existing methods that assume fixed sampling interval variation points, and establish a Wiener process-based random variation point detection mechanism. (3) Innovative time-varying dependency structure modeling. Future research should break through the limitations of current copula function selection, construct a dual degradation system lifespan prediction model integrating dynamic time-varying copula and hybrid copula structures, and enhance the mathematical characterization capability for complex dependency relationships.

## Figures and Tables

**Table 1:** Our common copula functions.

Copula Type	Copula Distribution Function C **(·)**
Gumbel	$\exp\left(-{\left({\left(-\ln F(X^{\left(1\right)})\right)}^{\alpha }+{\left(-\ln G(X^{\left(2\right)})\right)}^{\theta }\right)}^{1/\theta }\right)$
Frank	$-\frac{1}{\theta }\ln\left(1+\frac{\left(\exp(-\theta F(X^{\left(1\right)}))-1\right)\left(\exp(-\theta G(X^{\left(2\right)})-1\right)}{\exp(-\theta )-1}\right)$
Gaussian	$\phi_{2}\left(\phi^{-1}\left(F(X^{\left(1\right)})\right),\phi^{-1}\left(G(X^{\left(2\right)})\right);\theta \right)$
Clayton	$\max\left({\left(F{\left(X^{\left(1\right)}\right)}^{-\theta }+G{\left(X^{\left(2\right)}\right)}^{-\theta }-1\right)}^{-1/\theta },0\right)$

**Table 2:** 21 Sensor names and abbreviations.

Number	Symbol	Description
1	T2	Total temperature at fan inlet
2	T24	Total temperature at LPC outlet
3	T30	Total temperature at HPC outlet
4	T50	Total temperature at LPT outlet
5	P2	Pressure at fan inlet
6	P15	Total pressure in bypass-duct
7	P30	Total pressure at HPC outlet
8	Nf	Physical fan speed
9	Nc	Physical core speed
10	EPR	Engine pressure ratio (P50/P2)
11	Ps30	Static pressure at HPC outlet
12	PHI	Ratio of fuel flow to Ps30
13	NRf	Corrected fan speed
14	NRc	Corrected core speed
15	BPR	Bypass Ratio
16	farB	Burner fuel-air ratio
17	htBleed	Bleed Enthalpy
18	Nf_dmd	Demanded fan speed
19	PCNfR_dmd	Demanded corrected fan speed
20	W31	HPT coolant bleed
21	W32	LPT coolant bleed

**Table 3:** Trend values for the degradation of 21 sensors.

Number	1	2	3	4	5	6	7
trend value		0.6259	0.6715	0.7878			0.7197
number	8	9	10	11	12	13	14
trend value	0.4637	0.9418		0.8270	0.7443	0.4040	0.9270
number	15	16	17	18	19	20	21
trend value	0.2790		0.6915			0.7081	0.6766

**Table 4:** Modeling unknown parameter estimates.

Parameter	${\mu_{1}}^{\left(1\right)}$	${\sigma_{1}}^{\left(1\right)}$	${b_{1}}^{\left(1\right)}$	$\tau^{\left(1\right)}$
Value	2.7389 × 10^−3^	0.6597	2.0651	138
Parameter	${\mu_{2}}^{\left(1\right)}$	${\sigma_{2}}^{\left(1\right)}$	${b_{2}}^{\left(1\right)}$	
Value	0.0733	0.9896	1.9485	
Parameter	${\mu_{1}}^{\left(2\right)}$	${\sigma_{1}}^{\left(2\right)}$	${b_{1}}^{\left(2\right)}$	$\tau^{\left(2\right)}$
Value	3.2910 × 10^−3^	0.4003	2.1975	112
Parameter	${\mu_{2}}^{\left(2\right)}$	${\sigma_{2}}^{\left(2\right)}$	${b_{2}}^{\left(2\right)}$	
Value	0.1649	1.3337	1.5831	

**Table 5:** Copula function values and AIC results.

Copula Function	Gaussian	Frank	Gumbel	Clayton
θ value	0.9889	24.6423	1.6327	3.4860
Log-LF	1.6483 × 10^3^	509.4137	1.7994 × 10^3^	897.1463
AIC	−2.997 × 10^3^	−1.0048 × 10^3^	−3.5847 × 10^3^	−1.7803 × 10^3^

**Table 6:** AIC results for the four models.

	M1	M2	M3	M4
AIC	−3.5847 × 10^3^	−1.5526 × 10^3^	−1.6675 × 10^3^	−3.3536 × 10^3^

## Data Availability

Data were derived from a source in the public domain. The data used in this study are available at https://data.nasa.gov/Aerospace/CMAPSS-Jet-Engine-Simulated-Data/ff5v-kuh6 (accessed on: 10 December 2024).

## Abbreviations

The following abbreviations are used in this manuscript:

RUL	Remaining useful life
SIC	Schwarz information criterion
FHT	First hitting time
AIC	Akaike information criterion
PDF	Probability density function
CDF	Cumulative distribution function
IG	Inverse Gaussian
JDF	Joint distribution function
MDF	Marginal distribution function
